# Criterion validity of a competency-based assessment center in medical education – a 4-year follow-up study

**DOI:** 10.3402/meo.v19.25254

**Published:** 2014-09-12

**Authors:** Thomas Rotthoff, Martin S. Ostapczuk, Klaus D. Kröncke, Alexander Zimmerhofer, Ulrich Decking, Matthias Schneider, Stefanie Ritz-Timme

**Affiliations:** 1 Deanery of Study, Medical Faculty, Heinrich-Heine-University Duesseldorf, Duesseldorf, Germany; 2 Department for Endocrinology and Diabetes, University Hospital Duesseldorf, Duesseldorf, Germany; 3 Department of Orthopedics and Trauma Surgery, St. Josef Hospital, Moers, Germany; 4 Institute of Experimental Psychology, Heinrich-Heine-University Duesseldorf, Duesseldorf, Germany; 5 Institute of Biochemistry and Molecular Biology 1, Medical Department, Heinrich-Heine-University Duesseldorf, Duesseldorf, Germany; 6 ITB Consulting Ltd., Bonn, Germany; 7 Policlinic for Rheumatology, University Hospital Duesseldorf, Duesseldorf, Germany; 8 Institute for Forensic Medicine, University Hospital Duesseldorf, Duesseldorf, Germany

**Keywords:** assessment, educational, student selection, competency-based education, self-assessment, communication, assessment center

## Abstract

**Introduction:**

Core competencies have progressively gained importance in medical education. In other contexts, especially personnel selection and development, assessment centers (ACs) are used to assess competencies, but there is only a limited number of studies on competency-based ACs in medical education. To the best of our knowledge, the present study provides the first data on the criterion-related validity of a competency-based AC in medical education.

**Methods:**

We developed an AC tailored to measure core competencies relevant to medical education (social-ethical, communicative, self, and teaching) and tested its validity in *n*=30 first-year medical students using 3- to 4-year follow-up measures such as (a) objective structured clinical examinations (OSCE) on basic clinical skills (*n=*26), (b) OSCE on communication skills (*n=*21), and (c) peer feedback (*n=*18). The AC contained three elements: interview, group discussion, and role play. Additionally, a self-report questionnaire was provided as a basis for the interview.

**Results:**

Baseline AC average score and teaching competency correlated moderately with the communication OSCE average score (*r*=0.41, *p*=0.03, and *r*=0.38, *p*=0.04, respectively). Social-ethical competency in the AC showed a very strong convergent association with the communication OSCE average score (*r*=0.60, *p*<0.01). The AC total score also showed a moderate correlation with the overall peer feedback score provided in Year 4 (*r*=0.38, *p*=0.06). In addition, communicative competency correlated strongly with the overall peer feedback (*r*=0.50, *p*=0.02). We found predominantly low and insignificant correlations between the AC and the OSCE on basic clinical skills (*r*=−0.33 to 0.30, all *p*'s>0.05).

**Conclusion:**

The results showed that competency-based ACs can be used at a very early stage of medical training to successfully predict future performance in core competencies.

Competencies have progressively gained importance in international medical training and continuing medical education. In addition to competencies specific to the medical profession (i.e., diagnostic competence), core competencies, such as communication skills or social competency, are considered essential for ensuring that a student develops optimal professionalism (1). Eventually, the ability to integrate various competencies for optimal patient care is an important skill in the medical profession (2). That is why, ideally, a student's competency development should be continuously monitored and encouraged from the very beginning of medical training (3). In the present study, we developed elements of assessment aiming at the evaluation and feedback of core competencies (e.g., communicative, social-ethical, self, and teaching) of medical students based on the methods used in assessment centers (ACs). In the past decades, ACs have become increasingly popular (4). For the most part, their goal is the selection of suitable applicants for a professional carrier, that is, the prediction of future job performance for the purpose of personnel selection. Personnel development is their second major field of application. In ACs, participants work on different tasks in specific situations while being observed and evaluated by external raters. The formats of assessment vary – in-tray exercises, interview, role play, and group discussion constitute typical examples of AC elements. The prognostic success of an AC depends on how well the elements of the AC correspond to the demands of the later profession (5). Research has attested this method a good criterion-related predictive validity regarding future job performance (6–9). The results, however, are heterogeneous due to the various conceptions of AC, missing methodological standardization and predominantly low numbers of participants. Besides, ACs have often been criticized on methodological grounds for their low construct validity and inter-rater reliability as well as the varying number of assessment formats applied (10).

Although there is extensive literature on ACs in general, only a limited number of studies on the development of competency-based ACs in medical education have been published so far (11–14). This seems surprising as this approach is considered useful in the area of human resource development when, for example, the assessment of behavioral changes is of relevance (15). In the present study, we developed and conducted a competency-based AC in first-year medical students, that is, students way ahead of their professional careers. To investigate the criterion-related predictive validity of this AC, we conducted a longitudinal study assessing our students’ performance in other competency-based assessments over the course of 4 years of their medical training. To the best of our knowledge, the present investigation provides the first longitudinal data on the criterion-related validity of a competency-based AC in medical education.

## Methods

We developed an AC for the integrative assessment of core competencies relevant to medical education and tested its validity in first-year medical students. Longitudinal data using follow-up intervals of 3 and 4 years were obtained from other competency-based assessments, such as objective structured clinical examinations (OSCEs) and peer feedback.

### Demand analysis

In a first step, a working group consisting of the deans of study and faculty members along with psychologists from a consulting agency took part in a 1-day demand analysis dealing with the question what a medical student at our university should be like. For these properties, superordinate categories were defined, subsequently weighted, and assigned to the eight competencies of our competency-based curriculum: human-biological, preventive–diagnostic–therapeutic, communicative, social-ethical, scientific, self, teaching, and economic. Since the ideal number of competencies or behavioral dimensions in an AC is considered to be 2–4 (8), we focused on the four most relevant competencies which are not explicitly tapped by other examinations or assessment formats in the medical training at our university (see Table 1).

**Table 1:** Assignment of the categories identified by demand analysis to the competencies of the curriculum

Competence	Category
Social-ethical competency	Empathy, aptitude for teamwork, conflict handling skills, acceptance of ethical principles and social norms
Communicative competency	Sociableness, articulateness, appropriate body language, enthusiasm, persuasiveness
Self-competency	Organizational skills, strength of purpose, independence, emotional stability, resilience
Teaching competency	Analytical competency, ability to abstract, media literacy, didactic skills, interest in learning and teaching

In a next step, specific behaviors and attitudes were defined for the categories belonging to the four selected competencies, for example, ‘responds to objections of others, anticipates counter-arguments’ (communicative competency); ‘does not try to assert him/herself at the expense of others’ (social-ethical competency); ‘provides a complex issue simply and understandably without distorting it’ (teaching competency); and ‘is sure in his/her own decisions’ (self-competency). The behaviors were supposed to be relevant independently of the clinical context on the one hand, and relevant to the future job on the other hand. The design of the AC took the guidelines for creating an AC into account (7, 16, 17). Students were supposed to be observed in various tasks in the AC (see below), and the above-mentioned specific behaviors and attitudes were adopted to the various tasks, for example, ‘contributes to a pleasant atmosphere in conversations’ (social-ethical competency in the group discussion). The observed behaviors were scored on a scale from 1 (−) to 5 (+). In the recruitment phase, students in the second pre-clinical semester were contacted in a lecture and informed about the impending assessment day. Participation was voluntary. In the main, post-doctoral faculty members were recruited as raters by an information letter distributed to the university hospital and institutes.

### Questionnaire (Year 1)

An online questionnaire for competency testing had been developed. It served both as a self-assessment tool and as a basis for the interview which was to be conducted as one element of the AC. The questionnaire consisted of items pertinent to social-ethical, communicative, teaching competency, and self-competency. There were five questions for each of these competencies that were to be answered dichotomously (‘yes’=5 points or ‘no’=2 points). In the case of a positive answer, participants were asked for further clarification, for example, teaching competency: ‘Have you taken on a task in school, during your studies or in another situation, in which you had to instruct or teach someone? If yes: what was your task? How successful were you?’. In addition, students were asked to answer 15 statements per competency on a scale from ‘does not apply at all’ (1 point) to ‘fully applies’ (6 points), for example, communicative competency: ‘I strike the right note in nearly all situations’, or ‘I am good at expressing constructive criticism to those who have a different opinion’. Thus, a minimum score of 25 and a maximum score of 115 points could be obtained per competency – in total, the minimum questionnaire score was 100 and maximum 460 points.

### Elements of the AC (Year 1)

Despite the methodological difficulties of testing competencies individually in a meaningful way (2, 18, 19), we chose a ‘mixed method design’ for the AC, as this design is associated with a higher validity than a ‘one-instrument-to-one-competency’ approach. The choice of methods was based on two aspects: (a) applicability concerning the results of the demand analysis, and (b) practicability considering personnel and time resources. The following three elements were chosen: interview, group discussion, and role play. Interview and group discussion considered all four competencies, whereas the role play only took the social-ethical and communicative competency into account.

#### Structured interview

The interview took 30 min. Raters prepared for the interview by reading the results of the participant's questionnaire. In the interview, the answers to the questionnaire were discussed. Besides, raters had the opportunity to ask additional questions. The rating form contained behavioral anchors for the four competencies supposedly covered by the interview (see Table 1; positive example for social-ethical competency: ‘Gives examples of how she/he attends to the needs of others and their well-being’; negative example: ‘Reveals prejudice in comments about other people/groups’). Each of the four competencies was rated on a 5-point scale (1=‘has considerable gaps/shortcomings/problems/difficulties; does not meet the requirements’; 5=‘exceeds the requirements/expectations in all respects, outstanding’). In consequence, participants could achieve a minimum of 1 and a maximum of 5 points in each competency.

#### Group discussion

The group discussion lasted 45 min. The topic, chosen by the office of the dean, was ‘gene diagnostics’. The task was to develop practical recommendations as to how the potential benefits of gene diagnostics could be utilized while minimizing its risks. Again, raters used a rating form with behavioral anchors (positive example of self-competency: ‘Remains calm even in difficult phases of a conversation’; negative: ‘Picks up topics that have already been dealt with’). Behavior was rated on the 5-point scale described above.

#### Role play

This element of the AC consisted in a 5-min preparation phase which was immediately followed by a 15-min role play simulating a dialogue. The general topic was giving feedback to a fellow student. In this scenario, the rater assumed the part of the fellow student seeking feedback from the participant concerning him or her not having completed his part of a shared presentation. Social-ethical and communicative competencies were rated [examples for social-ethical competency: ‘Is sensitive to expressed and unexpressed needs of the conversation partner’ (positive); ‘Argues solely on a factual level and ignores the interpersonal level’ (negative)].

The questionnaire and the AC elements allowed for an assessment of the competencies to a different intensity.

### Procedure, raters, and participants

The raters (six physicians, two psychologists, and one specialist in German studies) were given a half-day training 3 days prior to the assessment day. The participating medical students (*n=*33) had already received an online questionnaire by e-mail. The questionnaires were completed electronically, responses were evaluated by a computer program, and the results were provided to the raters who were going to conduct the interviews. The raters were supposed to be able to ask probing questions in the interview based on the results of the questionnaire and the open comments. The entire assessment day lasted 8 hours. Each student was assigned to a different rater for each of the three AC elements. After completing the elements each student received personal feedback from one of the raters, usually the one who had conducted the interview, concerning his or her strengths and shortcomings in the various elements of assessment.

Feedback was based on the written documentation, that is, the rater's personal documentation of the interview and the other raters’ notes from group discussion and role play.

### Follow-up assessment (Year 3–4)

To assess the convergent and discriminant criterion-related predictive validity of the AC, AC scores were correlated with the students’ results in the following longitudinal assessments:

#### OSCE on basic clinical skills (Year 3)

In the third year of medical training at our university, students have to complete a summative and mandatory OSCE consisting of nine stations. Seven of the nine stations assess physical examination procedures, one station requires the insertion of a venous cannula and one station focuses on history taking. All nine stations are rated by a different rater on the basis of checklists. Participants can achieve a minimum of 0 and a maximum of 15 points per exercise. The summation of the nine scores per station results in the OSCE total score.

Note that apart from history taking, the remaining eight stations primarily assess practical skills. Yet even in the history taking exercise, structuring the history has priority, for example, providing an introduction, clarifying the objectives, and obtaining the complaint dimension such as localization and intensity. A maximum of 5 out of 15 points is awarded for non-verbal aspects such as empathy or emotionality.

#### OSCE on communications skills (Year 4)

At the end of the fourth year of study, our medical students have to complete another mandatory summative OSCE. The raters differed from the raters of the OSCE on basic clinical skills. In contrast to the third-year OSCE on basic clinical skills, the fourth-year OSCE was specifically designed to assess communicational skills required in challenging physician–patient encounters (20). The contents of the OSCE are anchored in our curriculum for communication in medical education (CoMeD) (21). At the time most students of the study entered the communication OSCE, the OSCE consisted of four stations requiring students to assume the role of a physician encountering actors trained as standardized patients. The four encounters focused on the ‘aggressive patient’, ‘breaking bad news’, ‘guilt and shame’, and ‘shared decision making’. In contrast to the OSCE on basic clinical skills, rating is based on global rating scales. Participants can achieve a minimum of 4 and a maximum of 20 points per station. The average communication OSCE score equals the sum of the single scores achieved in each station divided by 4. Due to a fundamental reform of our curriculum in 2013, the communication OSCE was allocated in another point of time within the curriculum. During the transition phase, several students completed the OSCE only with one station (‘breaking bad news’). In our study, six students were affected by this change (see below). To be able to use these students’ OSCE scores for further analyses, we used the score they achieved in the single station as a substitute of the OSCE average calculated for the remaining students.

#### Peer feedback (Year 4)

**Fig. 1:**
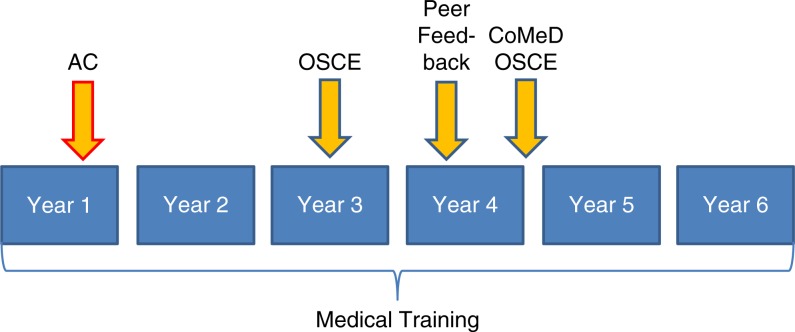
Chronological allocation of criterion-related measures. AC=assessment center; OSCE=objective structured clinical examination – basic clincial skills including history taking; CoMeD=Communication in Medical Education Duesseldorf – special communication skills.

In the fourth year of study, all students have the possibility to participate voluntarily in an anonymous peer feedback project. The project requires the participants to evaluate themselves and their peers in terms of communicative and social behavior. In our curriculum, students complete the whole fourth year in constant groups of 14 students. In the middle of the semester, students participating in the peer feedback project have to complete the self-assessment (10 items) and the assessment of each group member (10 items per group member) on a 5-point Likert scale (examples: 5=‘addresses the needs of others’ – 1=‘mostly tries to enforce his/her own needs’; 5=‘enriches class with instructive contributions’ – 1=‘contributes little to the enrichment of class’; 5=‘tries to understand other opinions’ – 1=‘is little tolerant towards other opinions’) (22). Self-assessment items are presented in the ‘I’ form, whereas peer assessment items are presented in the ‘he’ or ‘she’ form. Students can provide open comments to each item. The results of the self-assessment and the cumulated results of peer assessment are reported back to students by e-mail. If requested, every student has the opportunity to attend a personal counseling session. The overall self-assessment scores result from the summation of the 10 self-assessment items. The overall external assessment for each student is obtained by taking the mean of the assessments of participating group members. In our study, the self-assessment and external assessment scales showed an internal consistency (see below) of Cronbach's *α*=0.61 and 0.86, respectively (Fig. 1).

### Statistics

Statistical analyses were performed using the Statistical Package for the Social Sciences (SPSS) 17.0 for Windows. Effect sizes were determined with the program G*Power 3.1 (23). Results with *p*<0.05 were considered significant. Directed hypotheses were tested using a one-tailed significance level – if not noted otherwise, two-tailed significance levels were used.

Item discrimination and internal consistency (Cronbach's *α*) were determined to analyze the psychometric properties of the questionnaire. The part-whole-corrected discrimination index was considered very good for *r*≥0.50, acceptable for *r*≥0.30, and low for *r*≥0.10 (24). Cronbach's *α* was considered very good for *α*≥0.90, good for *α*≥0.80, acceptable for *α*≥0.70, barely acceptable for *α*≥0.60, and inacceptable for *α*<0.60 (24). For inference testing of dependent mean differences, the dependent-samples *t*-test (e.g., self-assessment vs. assessment by raters) or repeated-measures analysis of variance (ANOVA; e.g., social-ethical vs. communicative vs. teaching competency) was used. Mean differences between independent groups were tested using the independent-samples *t*-test (e.g., dropouts vs. non-dropouts). To correct for multiple comparisons in ANOVA, Bonferroni's correction was applied as a post-hoc test. Associations between two continuous variables were calculated using Pearson's correlation coefficient (*r*). Differences between two correlation coefficients were assessed for significance using a *z*-test by Meng et al. (25) implemented in the new freely available program cocor (26). We followed Cohen's suggestion (27) and determined effect sizes for all statistical tests. According to Cohen, for the effect size measure *d* in *t*-tests, it holds that *d*≥0.80=large effect, *d*≥0.50=moderate effect, and *d*≥0.20=small effect. For *η* ^2^ derived from ANOVA, *η* ^2^≥0.14 implies a large effect, *η* ^2^≥0.06 a moderate and *η* ^2^≥0.01 a small one. The correlation coefficient *r* is itself a measure of effect size, with *r*≥0.50=large, *r*≥0.30=moderate, and *r*≥0.10=small.

## Results

### Sample

A total of 33 students (24 female) participated in the study, of whom 29 (20 female) completed the questionnaire, 30 (22 female) completed elements of the AC, and 26 (17 female) completed both the questionnaire and the AC.

Of the original sample, two students (two female) had quit their medical training before entering Year 3, while three students (three female) had not passed the first state examination which is a prerequisite for entering Year 3. These five dropouts achieved descriptively lower scores both on the questionnaire and in the AC, but none of the mainly small to moderate differences achieved statistical significance (all *p*'s>0.05). Some further students had passed the first state examination behind schedule, which is why they had not entered Year 3 and 4, respectively, by the time data were analyzed. Besides, participation in the peer feedback project was voluntary. In consequence, follow-up results were available as follows:

1. OSCE on basic clinical skills (Year 3): *n*=26 (18 female),
2. OSCE on communication skills (Year 4): *n*=21 (15 female), 6 of these students (all female) participated in the reduced one-exercise-OSCE,
3. Peer Feedback (Year 4): *n*=13 (self-assessment, 10 female) and *n*=18 (peer assessment, 14 female), respectively.

### Questionnaire

To assure comparability between questionnaire and AC, percentages were calculated both for the questionnaire and the AC dimensions. On average, students achieved 72.9% of the maximum score in the questionnaire (*M*=335.59, SD=27.27, out of 460 points). There were significant differences between participants’ self-reported competencies [*F*(3,84)=3.36, *p*=0.02, *η* ^2^=0.11]: Students rated themselves significantly higher in teaching competency (87.07 points/75.7%) than in communicative competency (81.00 points/70.4%; *p*<0.01). The remaining competencies did not differ significantly (see Table 2).

**Table 2:** Self-reported competency scores and competency scores achieved in assessment center

Questionnaire (Q) (*n=*29)	Mean (% of potential maximum)	SD	Minimum	Maximum
Q – Social-ethical competency	83.07 (72.2)	9.65	56	101
Q – Communicative competency	81.00 (70.4)	9.68	63	100
Q – Self-competency	84.45 (73.4)	10.55	63	102
Q – Teaching competency	87.07 (75.7)	7.44	71	103
**Q – Total score**	**335.59 (72.9)**	**27.26**	**287**	**399**

Assessment center (AC) (*n=*30)				

AC – Social-ethical competency	3.95 (79.0)	0.55	2.5	5.0
AC – Communicative competency	3.96 (79.2)	0.50	3.0	5.0
AC – Self-competency	4.01 (80.2)	0.49	3.0	5.0
AC – Teaching competency	3.65 (73.0)	0.63	2.0	5.0
**AC – Average score**	**3.95 (79.0)**	**0.44**	**2.9**	**4.9**
AC – Interview	3.94 (79.0)	0.63	2.8	5.0
AC – Group discussion	3.81 (76.2)	0.65	2.8	5.0
AC – Role play	4.08 (81.6)	0.72	3.0	5.0

SD=standard deviation.

Questionnaire (Q): minimum score=100, maximum score=460. Assessment center (AC): minimum score=1, maximum score=5.

Total and average scores, respectively, are printed in bold.

Psychometric analyses of the questionnaire revealed that one dichotomous item could not be considered for the computation of part-whole corrected discrimination due to a lack of variance, since all students agreed. Of the remaining 19 dichotomous items, 14 items showed acceptable to very good discrimination indices. Only six of the 60 continuous items were of insufficient discrimination. Reliability analyses using Cronbach's *α* yielded a good overall reliability of *α*=0.85 (79 items), with *α*=0.63 for the 19 dichotomous items and *α*=0.86 for the 60 continuous items.

### Assessment center

Overall, raters gave rather positive ratings (on average >3.50 on a scale from 1 to 5 and >70%, respectively). Like in the questionnaire, there were significant differences between the competency ratings [*F*(3,87)=5.51, *p*<0.01, *η* ^2^=0.16]: With ratings being similarly high for social-ethical, communicative, and self-competency (ranging from 3.95 to 4.01 points and 79.0 to 80.2%, respectively), teaching competency (3.65 points/73.0%) was rated significantly worse than the three remaining competencies (all *p*'s<0.05). As to the three AC elements, there were no significant differences between the ratings [3.81 to 4.08 and 76.2–81.6%, respectively; *F*(2,58)=1.43, *p*=0.25, *η* ^2^=0.05; see Table 2].

To determine whether participants assessed themselves more strictly via questionnaire than they were externally assessed by the raters in the three AC elements, we calculated dependent *t*-tests. Pooled across the four competencies, students’ self-rating was not significantly poorer [*t*(25)=−0.78, *p*=0.44, *d*=0.15]. Separate analyses, however, revealed significantly lower self-ratings in comparison to the raters’ assessment on all competencies (all *p*'s<0.05, moderate effect sizes from *d*=0.50 to 0.67) except for teaching competency [*t*(25)=0.90, *p*=0.19, *d*=0.18]. Interestingly, none of the competencies showed a significant correlation between self-report (questionnaire) and external assessment [AC; *r*'s=0.15 to 0.26, all *p*'s >0.05 (one-tailed)].

To examine which of the three AC elements constitutes the best predictor of overall AC performance, we computed Pearson correlations between the three elements and the overall AC score. The best predictor of overall performance was the role play, which correlated best with overall performance [*r=*0.77, *p<*0.001 (one-tailed)], followed by group discussion [*r*=0.65, *p*<0.001 (one-tailed)] and the interview [*r=*0.57, *p<*0.001 (one-tailed)]. However, the three correlations did not differ significantly (all *p*'s>0.05). As to the competencies, social-ethical [*r*=0.83, *p*<0.001 (one-tailed)] and communicative competency [*r*=0.82, *p*<0.001 (one-tailed)] showed the strongest associations with the AC total score, followed by teaching competency [*r*=0.77, *p*<0.001 (one-tailed)], and self-competency [*r*=0.62, *p*>0.001 (one-tailed)]. These correlations did not differ significantly either (all *p*'s>0.05).

Construct validity of the AC was examined by correlating the corresponding competencies between the different elements within the AC. The resulting correlations were rather low and insignificant (*r*'s=−0.29 to 0.21), except for the moderate correlation between social-ethical competency as assessed by group discussion and social-ethical competency as assessed by interview [*r*=0.32, *p*=0.04 (one-tailed); see Table 3].

**Table 3:** Pearson correlations (*r*) between competencies within assessment center

(*n*=30)									

Social-ethical competency			Communicative competency			Self-competency		Teaching competency	

	Interview	Group discussion		Interview	Group discussion		Interview		Interview
Group discussion	0.32*		Group discussion	−0.04		Group discussion	−0.29†	Group discussion	0.17
Role play	0.21	0.05	Role play	0.00	0.14				

† *p*<0.10 (one-tailed)

* *p<*0.05 (one-tailed).

Significant correlations are printed in bold.

### Criterion-related predictive validity

An examination of the third-year follow-up measures (OSCE on basic clinical skills) revealed that neither the OSCE total score nor the OSCE station ‘history taking’ correlated significantly with the AC (see Table 4). The correlations between the AC and the eight remaining OSCE stations (*r*=−0.33 to 0.30, all *p*'s>0.05), rather examining technical medical skills than social-communicative core competencies, were predominantly low and insignificant.

**Table 4:** Pearson correlations (*r*) between questionnaire scores, elements of assessment center and follow-up assessment

		Questionnaire – total score		AC – interview	AC – group discussion	AC – role play	AC – average score	AC – social-ethical competency	AC – communicative competency	AC – self-competency	AC – teaching competency
History taking (OSCE exercise)	(*n*=24)	−0.03	(*n*=26)	0.18	0.10	−0.10	0.08	0.15	0.07	−0.14	0.27
OSCE (total score)		−0.02		0.19	−0.11	0.07	0.07	0.02	0.20	−0.06	−0.02
CoMeD OSCE (average score)	(*n*=21)	0.34†	(*n*=21)	0.24	0.32†	0.29	0.41*	0.60**	0.17	−0.03	0.38*
Peer feedback (self-assessment)	(*n*=12)	0.41†	(*n*=13)	0.47†	−0.37	−0.39	−0.14	−0.13	0.12	0.19	−0.28
Peer feedback (external assessment)	(*n*=18)	−0.09	(*n*=18)	0.29	0.23	0.24	0.38†	0.19	0.50*	0.15	0.35†

AC=assessment center; CoMeD=Communication in Medical Education Düsseldorf; OSCE=objective structured clinical examination.

Significant correlations are printed in bold.

† *p*<0.10 (one-tailed)

* *p*<0.05 (one-tailed)

** *p*<0.01 (one-tailed).

As to the fourth-year measures, the AC average score and teaching competency correlated moderately with the communication OSCE average score [*r*=0.41, *p*=0.03, and *r*=0.38, *p*=0.04 (one-tailed), respectively]. Social-ethical competency showed a very strong convergent association with the communication OSCE average score [*r*=0.60, *p*<0.01 (one-tailed)]. All three AC elements correlated consistently with the communication OSCE (*r*=0.24 to 0.32), these low to moderate correlations, however, did not attain statistical significance (all *p*'s>0.05).

The AC total score also showed a moderate and marginally significant correlation with the overall external peer feedback score provided in Year 4 [*r*=0.38, *p*=0.06 (one-tailed)]. In addition, communicative competency correlated strongly with the overall peer feedback [*r*=0.50, *p*=0.02 (one-tailed)]. Interestingly, the pattern of results was somewhat different for self-assessment within the peer feedback project, which did not correlate with the total AC score, yet marginally significantly with the interview score [*r*=0.47, *p*=0.05 (one-tailed); see Table 4]. The low and insignificant correlation between the external peer feedback score and the self-assessment [*r*=0.24, *p*=0.21 (one-tailed)] emphasizes the discrepancy between self-report and other report.

Note that questionnaire scores did not correlate significantly with any of the criterion-related measures, although it showed some moderate, yet insignificant associations with the communication OSCE average score [*r*=0.34, *p*=0.06 (one-tailed)] and peer feedback self-assessment [*r*=0.41, *p*=0.09 (one-tailed); see Table 4]. In sum, the pattern of criterion-related associations reveals that, in contrast to the questionnaire, the AC is particularly predictive of the communicational skills as assessed by the fourth-year communication OSCE and the communicative, social, and teaching behaviors assessed by the external fourth-year peer feedback. Of the AC elements, the interview provides the strongest, yet insignificant positive correlations with convergent follow-up measures (ranging from *r*=0.24 to 0.47). As to the competencies, the pattern of results is mixed: Each competency has its merits – except for self-competency which does not correlate significantly with any single criterion-related measure.

## Discussion

In the present investigation, an AC tailored to measure core competencies relevant to medical education was tested for its validity in first-year medical students using longitudinal data among others.

The results show that such an AC can indeed fruitfully assess competencies different from mere practical skills or medical knowledge: The dimensions of the present AC, especially the social-ethical, communicative and teaching competency, were well suited to predict medical students’ social and communicative competencies – as represented by a communication OSCE and peer feedback – over a period of 4 years. Contrary to the moderate to strong convergent correlations with the communication OSCE and peer feedback, correlations between AC and the basic clinical skills OSCE, including history taking, were consistently low and insignificant. This finding is not surprising, it rather shows the discriminant validity of our AC, bearing in mind that eight of the nine OSCE stations assessed practical medical skills only. And even taking a good history – at least as defined by our OSCE – rather requires a specific structure guided by medical knowledge than purely communicative or social skills. In line with previous literature on ACs in general, the present AC thus showed good convergent and discriminant criterion-related predictive validity in contrast to a weaker construct validity, as indicated by the low to moderate correlations of the corresponding competencies between the different elements in the AC.

Apart from their low construct validity, ACs have often been criticized for their high costs and low standardization among others (4, 10). It might therefore seem attractive to replace time-consuming AC elements with more cost-effective and standardized instruments such as questionnaires. In spite of its good psychometric quality and the fact that it was supposed to assess the same competencies as the AC, the questionnaire used in the present study did not correlate with any of the criterion-related measures significantly though. It showed the highest, yet still moderate and insignificant correlation with self-assessment within the peer feedback (*r*=0.41), that is, another self-report measure, which in turn strongly differed from the external peer feedback. This finding is noteworthy given that the questionnaire served as a basis for the interview which, on the other hand, of the three AC elements co-varied most consistently with the convergent criterion-related measures (small to moderate correlations of *r*=0.24–0.47). This pattern of results, that is, the low correlations between the questionnaire and the criteria as well as the discrepancies between self- and other-assessment in peer feedback, supports the notion that even well-conceived, standardized self-report measures cannot replace an AC based on external behavioral observations, but may be well suited to complement or prepare for such an AC. This recommendation seems in line with findings on people's limited ability to accurately self-assess (28, 29). We are, however, not implying that self-assessment is not valid at all, but rather emphasizing that self-reports must be treated with caution and should be completed and validated by external observations (or vice versa), such as the formats used in the present study (AC, OSCE, peer feedback).

Regarding the results in more detail, the AC total score correlated positively with the communication OSCE and the external assessment of the peer feedback. Of the competencies, especially the social-ethical and communicative competency, followed by teaching competency, proved to be good predictors of future behavior. It seems surprising though that communicative competency did not correlate significantly with the communication OSCE, as both formats explicitly concentrate on communicative aspects. Note, however, that the communication OSCE focuses on very specific aspects of the physician–patient encounter, while the AC communicative competency taps rather general communicative aspects, such as responding to objections of others, keeping eye contact, speaking precisely, or a positive body language (see Table 1). This might also be the reason for the high correlation between communicative competency and the external peer feedback. In contrast to that, self-competency did not correlate significantly with any criterion. This probably prods to the intrinsic difficulty of conceiving observable behaviors tapping self-competency. Apart from this possibly general problem, the competencies as assessed by different AC elements did not correlate significantly with each other, except for social-ethical competency as measured by interview and group discussion. In literature, the sometimes low differentiability between dimensions, that is, competencies in the present AC, has been widely and critically discussed (4, 10). The critics argue that the design of the AC and its development are responsible for its limited construct validity. A reduction of the number of dimensions, here competencies (8, 9), as well as a second rater per AC element might have increased construct validity in the present study (9). Note, however, that despite some absent expected construct- and criterion-related correlations, the competencies showed a differential pattern of associations with the criterion-related measures – with some of them predicting behaviors over a period of 4 years very well, for example, social-ethical competency and communication OSCE (*r*=0.60) or communicative competency and peer feedback (*r*=0.50). In other words, a medical student who performs poorly in an AC in the first year of study does not perform much better with patients or fellow students 4 years later – a fact which fellow students are perfectly aware of, even though the person concerned does not see this as a problem. It is probably not far-fetched to predict that such a student will eventually become a physician with weaker communicative and social competencies. These results show that a differentiated view on different competencies within an AC may provide interesting insights even if results in terms of construct validity are disillusioning. Specifically, such an AC can be used both for student selection and for student development at an early stage of medical training. The implementation of such an AC requires high personal and financial efforts. In consequence, each faculty has to reconsider how much importance it attaches to competencies other than mere medical skills and how much the faculty is willing (or able) to invest in the promotion of those.

Finally, we would like to acknowledge some limitations of the study: (a) the sample size was small and not all participants took part in all parts of the assessment for personal reasons (e.g., schedule conflicts) which diminishes the statistical power and general applicability of our results. (b) There were dropouts in the follow-up assessments, since some students quit their medical training or did not pass the first state examination – either in time or at all. (c) Not all raters took part in all elements of the assessment. Consequently, comparability was reduced and inter-rater reliability could not be computed. (d) Female students were overrepresented in our sample. The proportion (approximately 70%), however, does not deviate from the actual proportion of female medical students at our university and Germany in general. (e) Selection bias might have been responsible for the mild external ratings in the AC compared to students’ self-assessment in the questionnaire: Since participation in the AC was voluntary, participating students might have been particularly motivated and therefore highly assessed by the raters.

In sum, the results of the – to the best of our knowledge – first study of an AC in medical education using a follow-up interval of 4 years show that competency-based ACs can be used at a very early stage of medical training to successfully predict future performance in medical training and possibly as a physician.

## Ethical approval

Ethical approval was given by the independent Ethics Committee of the Medical Faculty (Study no. 3851).
